# An Investigation of Surface Corrosion Behavior of Inconel 718 after Robotic Belt Grinding

**DOI:** 10.3390/ma11122440

**Published:** 2018-12-02

**Authors:** Junwei Wang, Jijin Xu, Xiaoqiang Zhang, Xukai Ren, Xuefeng Song, Xiaoqi Chen

**Affiliations:** 1 Shanghai Key Laboratory of Materials Laser Processing and Modification, School of Materials Science and Engineering, Shanghai Jiao Tong University, Shanghai 200240, China; junwei12163@126.com (J.W.); zxq907739436@163.com (X.Z.); renxukai@126.com (X.R.); songxfeng@sjtu.edu.cn (X.S.); 2 Department of Mechanical Engineering, University of Canterbury, Christchurch 8140, New Zealand

**Keywords:** robotic belt grinding technique, corrosion behavior, nickel-based superalloy, roughness, residual stress

## Abstract

Surface corrosion resistance of nickel-based superalloys after grinding is an important consideration to ensure the service performance. In this work, robotic belt grinding is adopted because it offers controllable material processing by dynamically controlling process parameters and tool-workpiece contact state. Surface corrosion behavior of Inconel 718 after robotic belt grinding was investigated by electrochemical testing in 3.5 wt % NaCl solution at room temperature. Specimens were characterized by morphology, surface roughness and residual stress systematically. The potentiodynamic polarization curves and electrochemical impedance spectroscopy (EIS) analysis indicate the corrosion resistance of the specimen surface improves remarkably with the decrease of abrasive particle size. It can be attributed to the change of surface roughness and residual stress. The energy dispersive X-ray spectroscopy (EDS) indicates that niobium (Nb) is preferentially attacked in the corrosion process. A plausible electrochemical dissolution behavior for Inconel 718 processed by robotic belt grinding is proposed. This study is of significance for achieving desired corrosion property of work surface by optimizing grinding process parameters.

## 1. Introduction

Inconel 718 superalloy has been widely utilized in gas turbine engineering, submarine, nuclear reactors, oil and gas production parts because of its good service performance [1,2,3]. For ship gas turbine and submarine which mainly work in the marine environment, the corrosion behavior of Inconel 718 must be fully understand to adopt effective countermeasures ensuring the safety of the alloy system. In addition, a good corrosion resistance is beneficial to the service life of the parts because most cracks commence at the surface of the material. Corrosion performance is mainly affected by material compositions [4] and surface states such as surface roughness and surface stress [5]. The high nickel content of Inconel 718 makes the alloy have a relatively strong resistance to chloride stress cracking corrosion. At the same time, due to the presence of chromium, corrosion resistance is even better than pure nickel in the oxidizing environment. Wang et al. [6] investigated the corrosion behavior of Inconel 718 in electrochemical machining, suggesting that the generated niobium carbide and niobium oxide have an important influence on the corrosion process of nickel-based superalloy. Jebaraj et al. [7] reported hydrogen permeation of Inconel 718 in different states and found that the hydrogen trapping in cold rolled and precipitation hardened Inconel 718 is irreversible. These studies objectively analyze the chemical elements influencing the corrosion performance of the alloy. Additional effective measures need to be devised to improve corrosion performance.

In order to enhance the corrosion resistance of nickel-based superalloy, various processing technologies were globally employed. Karthik et al. [8] reported the enhanced corrosion performance in Inconel 600 through laser shock peening without coating. They found the larger and deeper compressive residual stresses and smaller surface roughness are the main reasons for the increased corrosion performance. However this study was conducted on Inconel 600, and was not supported by an actual corrosion experiment, such as immersion tests. Huang et al. [9] investigated the electrochemical corrosion behavior of Inconel 718 sheets treated by electron beam welding in 3.5 wt % NaCl solution. To reduce the adverse impact on corrosion performance, they adopted a method of subsequent heat treatment. Khan et al. [10] studied the corrosion performance of Inconel 718 in the simulated body fluid. The results show that laser surface-modified process can improve the corrosion resistance of materials effectively. Akyol et al. [11] deposited Ni-P-W-CNF (Carbon Nanofibers) composites on the works by electroless method, and obtained a good wear and corrosion resistance. However, this improvement approach causes pollution. Narayanan et al. [12] analyzed the change of impingement angle to compare the corrosion resistance of nickel-based superalloy which was laser surface treated. The corrosion performance was increased by about 1.5 times due to the minimized energy transfer of laser. It is a feasible process to improve erosion resistance under the premise of ensuring efficiency. Arrizubieta et al. [13] combined laser material deposition and laser surface processes for the complete manufacturing of Inconel 718 components. The surface quality was improved and the roughness was reduced. Nevertheless, the process is relatively complicated. Karthik et al. [5] reviewed laser peening without protective coating technology as a mechanical surface modification method. The method can significantly improve the corrosion resistance of metallic materials due to the factors including surface roughness and compressive residual stress. These studies analyze the corrosion properties and influencing factors of nickel-based alloys under different conditions. However, methods to improving corrosion resistance require additional processing, such as material deposition, laser peening, etc. These measures not only consume time and resources, but also inevitably increase production costs. It is desired that a robotic belt grinding process could improve the corrosion performance by optimizing its process parameters and without additional processing.

Robotic belt grinding offers controllable material processing by dynamically controlling process parameters and tool-workpiece contact state, which is otherwise difficult to achieve by manual grinding [14]. While conventional CNC grinding machine offers position control, a robotic grinding system can readily incorporate both position and force control. As a result, desired profile finishing accuracy and surface properties can be obtained by online tool condition monitoring and optimal process control [15,16]. As such, we have adopted robotic belt grinding in this study.

Pradhan et al. [17] analyzed the influence of surface roughness on corrosion behavior of Inconel 718 in a simulated marine environment at high temperature for a relatively long time. The results show that the higher roughness increases the surface area for the corrosion behavior and reduces the corrosion resistance of the works. However, roughness is only one of the contributing factors affecting corrosion resistance. Based on our previous study [18], we found that the finished surface obtains a considerable residual stress, which has a significant impact on surface corrosion performance. This phenomenon, which is neither fully understood nor discussed by existing works, is investigated in detail in this paper. By controlling appropriate parameters, the robotic belt grinding process can achieve the ideal compressive residual stress in the sub-surface and lower surface roughness, which improves surface corrosion performance. To the best of our knowledge, the mechanism of residual stress affecting corrosion Ni-based superalloys has not been reported elsewhere.

Tressia et al. [19] investigated the influence of abrasive particle size and different aqueous solution on the abrasive wear mechanisms in the grinding process. Turnbull et al. [20] reported the sensitivity of stress corrosion cracking of stainless steel to surface machining and grinding procedure. The above research works mostly based on manual grinding suffer from their uncontrollability and inconsistency. Robotic belt grinding is a relatively new precision machining technique, and has attracted great attention for its advantages over conventional manual grinding procedure [21,22]. Although great progress on the corrosion behavior of nickel-based superalloy has been made, the related research on surface corrosion behavior and influencing factors of nickel-based superalloy after robotic belt grinding has been barely reported.

In this work, electrochemical corrosion of Inconel 718 was investigated to evaluate the corrosion behavior of the alloy under robotic belt grinding. The experiment was carried out in 3.5 wt % NaCl solution, which is a commonly used corrosive solution in electrochemical corrosion experiments [23]. The potentiodynamic polarization curves and electrochemical impedance spectra (EIS) were measured. Furthermore, the corroded specimens were examined by scanning electron micrograph (SEM) and energy dispersive X-ray spectroscopy (EDS). The influencing factors including surface roughness and residual stress were discussed in detail. 

## 2. Materials and Methods

### 2.1. Materials

The nickel-based superalloy Inconel 718 used in this study was provided by Baoshan Iron & Steel Co., Ltd. (Shanghai, China). The Inconel 718 was hot forging state, and annealed at 980 °C for 10 min followed by air cooling. Then, the materials were acid pickled. The acid used is a mixed solution, whose concentration is 10 vol % HNO_3_ + 7 vol % HF. The purpose of acid pickling is to remove the oxide formed on the surface of the material during heat treatment. Its chemical compositions were investigated by inductively coupled plasma-optical emission spectroscopy (ICP-OES) and the results are listed in Table 1.

The as-received material was cut into square bars with dimensions of 15 mm × 15 mm × 500 mm using electric discharge machine wire cutting. The annealing process can improve the ductility and formability, which is meaningful for the subsequent processing.

### 2.2. Experiment Set-Up

**Figure 1:**
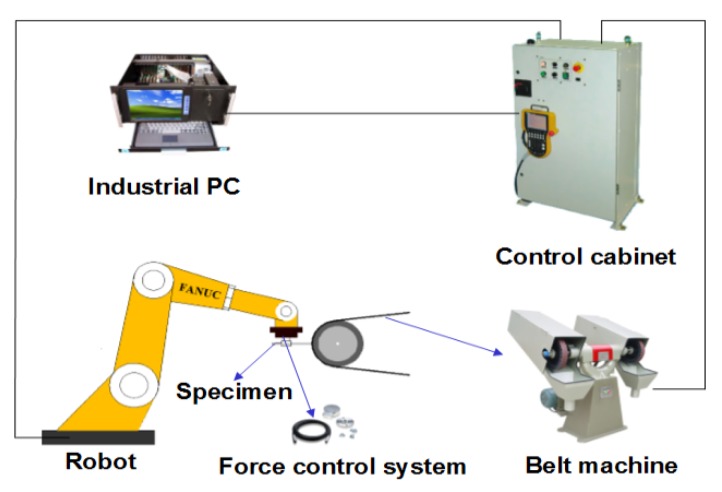
Schematic diagram of experimental setup.

The robotic belt grinding system used in this study mainly consists of four components which are control cabinet, industrial PC, FANUC (Fuji Automatic Numerical Control) robot with a force control sensor and a belt machine, as shown in Figure 1. The abrasive belt was supplied by 3 M China Limited, and its abrasive particles is special Al_2_O_3_ stocked on an elastic paper strip reinforced with fibers. 

The prepared specimens were ground by the optimum processing parameters of grinding force 178 kPa and belt speed 21 m/s. Three kinds of belt with different particle size were selected, which are 36, 80 and 120 M, corresponding to grain sizes of about 500, 178, and 125 μm, respectively. The mesh number is inversely proportional to the particle size. The larger the mesh is, the smaller the abrasive particle. Therefore, there are three sets of parameters in this experiment: 36 M with 178 kPa and 21 m/s, 80 M with 178 kPa and 21 m/s, 120 M with 178 kPa and 21 m/s.

### 2.3. Test Methods

After the grinding process, a series of tests were carried out. The surface roughness of the ground specimens was tested with a roughness tester (SJ-410, Mitutoyo, Kawasaki, Japan) and the measuring direction was perpendicular to the grinding direction. The residual stress states on the ground surface were measured by an X-ray Stress Analyzer (LXRD, Proto, Sacramento, CA, USA) with Mn-K_α_ radiation and Cr filter, using standard sin^2^ ψ method under 18 kV (voltage) and 4 mA (current). The (311) plane with a 2θ of 165.32 was chosen as the shifts of diffraction peaks.

To evaluate the corrosion resistance of the alloy, electrochemical testing was employed at room temperature in 3.5 wt % NaCl solution and free air. The samples were held in corrosive solution for 65 min, including 20 min of immersion time and 45 min of corrosion time. It was conducted using a CHI 660E electrochemical system with Saturated Calomel Electrode (SCE), Pt wire and the ground specimen as reference, auxiliary and working electrode respectively. The dimension of the test specimen was 10 mm × 10 mm with an area of 100 mm^2^. Potentiodynamic polarization testing was performed over the applied potential ranging from −1.2 V to 1.5 V at a sweep rate of 1 mV/s. Each potentiodynamic polarization measurement was repeated three times to obtain a reliable result. Tafel extrapolation method was used to calculate the corrosion potential (E_corr_) and corrosion current (I_corr_). EIS testing was conducted with the same device under the same condition. The perturbation voltage is 10 mV. The frequency ranges from 100 kHz to 10 mHz. The EIS test was carried out at open circuit potential (E_corr_). EIS test was also repeated three times.

Immersion tests were also conducted after the grinding process. The ground specimens of 15 mm × 15 mm were rinsed carefully by acetone, ethanol and distilled water in turn. The immersion tests were carried out in 3.5 wt % NaCl solution at room temperature. Corrosion products were removed by chemical cleaning with 10 vol % HNO_3_ + 7 vol % HF. The mass loss of the specimens were measured by an analytical balance (FA124, Sunny Hengping, Shanghai, China) with a precision of 10 μg. Each mass loss was measured for three times to obtain a reliable result.

Morphology characterization was accomplished using a JSM-7600F field-emission scanning electron microscope (SEM, JEOL, Akishima, Japan) at the accelerating voltage of 5 kV and probe current of 2 × 10^−10^ A. The SEM machine was equipped with an energy dispersive X-ray spectroscopy (EDS) to identify the compositions of specimen surface. The accelerating voltage is 20 kV and the working distance is 15 mm.

## 3. Results and Discussion

### 3.1. Electrochemical Analysis

**Figure 2:**
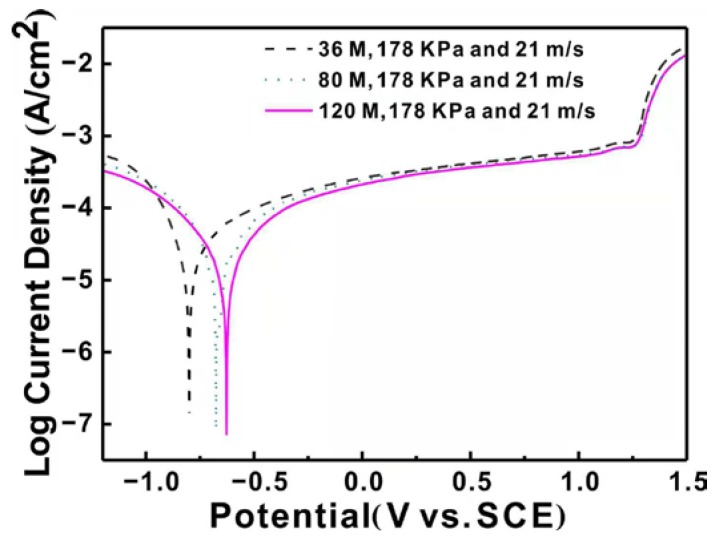
Potentiodynamic polarization curves of the ground surface with different abrasive particle sizes. Saturated Calomel Electrode (SCE).

Figure 2 shows potentiodynamic polarization curves of the ground surface with different abrasive particle sizes. The potentiodynamic polarization curves exhibit similar shape for the three specimens and there are obvious passivation regions in accord with the current response [24]. The current density has an increasing trend as the applied anodic voltage increases. In working electrodes, there exists a cathodic reduction process, which is a hydrogen evaluation reaction [25]. 

In the anodic regions, there is a typical active-passive-trans-passive behavior, displaying a limiting current density with the increase of the corrosion potential. This is owing to the existence of the compact oxidation film on the ground surface [26]. The passivation region begins near the corrosion potentials and there is a rapid increase of current density when the applied potential increases to a certain value around 1.25 V due to the dissolution of the oxidation film. In addition, the pit corrosion occurs according to the steep increase of the current. Simultaneously, the oxidation reaction of Inconel 718 proceeds, leading to a passivation oxidation film on the specimen surface. 

To compare the corrosion performance of the ground specimens clearly, corrosion current density (I_corr_) and corrosion potential (E_corr_) are calculated from the potentiodynamic polarization curves [27], as presented in Table 2. The corresponding errors are also listed and have a good repeatability. It can be found that corrosion potential (E_corr_) increases forwards the positive direction and the corrosion current density (I_corr_) decreases with the decrease of the particle size. It is well known that the more positive the corrosion potential is, the higher corrosion resistance [28]. In addition, the corrosion property is also influenced by corrosion rate, which can be represented by the corrosion current density (I_corr_) according to Faraday’s law [29]. The specimen ground by particle size of 120 M shows the highest corrosion potential and the lowest corrosion current density, indicating the best corrosion property. From the analysis above, it can be concluded that the corrosion property of the specimen surface increases remarkably with the decrease of particle size. 

**Figure 3:**
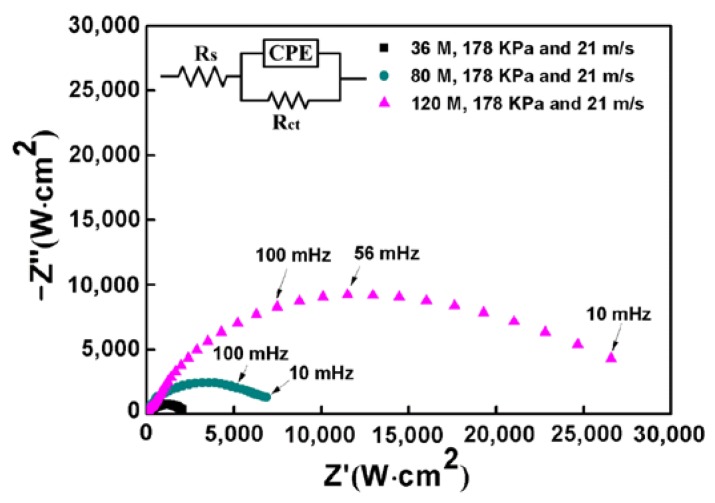
Nyquist plots of the ground surface with different abrasive particle sizes. Constant phase elements (CPE).

In order to further understand the electrochemical corrosion behavior of Inconel 718 treated by robotic belt grinding, EIS testing was carried out. Typical Nyquist plots of the specimens are shown in Figure 3. There is an obvious capacitance loop, because the corrosion process is dominated by the charge transfer step. The capacitance loop is not a standard semicircle due to the heterogeneous corrosion surface [30]. The diameter of capacitance loop increases with the decrease of abrasive particle size. The value of the diameter represents the corrosion resistance of the test specimen [31]. Samples ground using larger particle sizes have better corrosion performance [32]. It can be concluded that a smaller abrasive particle size is beneficial to the corrosion resistance of the specimen surface. The results of EIS testing are consistent with the potentiodynamic polarization tests well.

**Figure 4:**
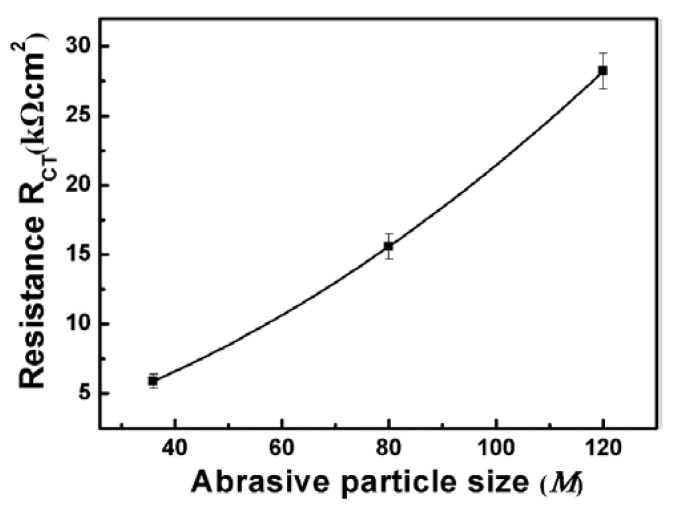
R_CT_ of the equivalent circuit evaluated by fitting the impedance spectra of the ground surface with different abrasive particle sizes.

To further explain the corrosion behavior, an equivalent circuit diagram was used, as shown in the insert of Figure 3. The R_S_ represents the solution resistance, R_CT_ represents charge transfer resistance at the interface. Moreover, pure capacitors were substituted by constant phase elements (CPE) and the impedance of CPE was defined by equation: $Z_{CPE}={\left[Q{\left(j\omega \right)}^{n}\right]}^{-1}$, where Q is the frequency independent constant; n is the exponential coefficient, the value of which is between 0 and 1; j is an imaginary unit and ω is an angular frequency [33]. The fitted resistance values R_CT_ were listed in Figure 4. It could be found that the R_CT_ increases as the abrasive particle size decreases, indicating that the corrosion resistance increases with the decrease of abrasive particle size in NaCl solution. 

### 3.2. Corrosion Morphology Analysis

**Figure 5:**
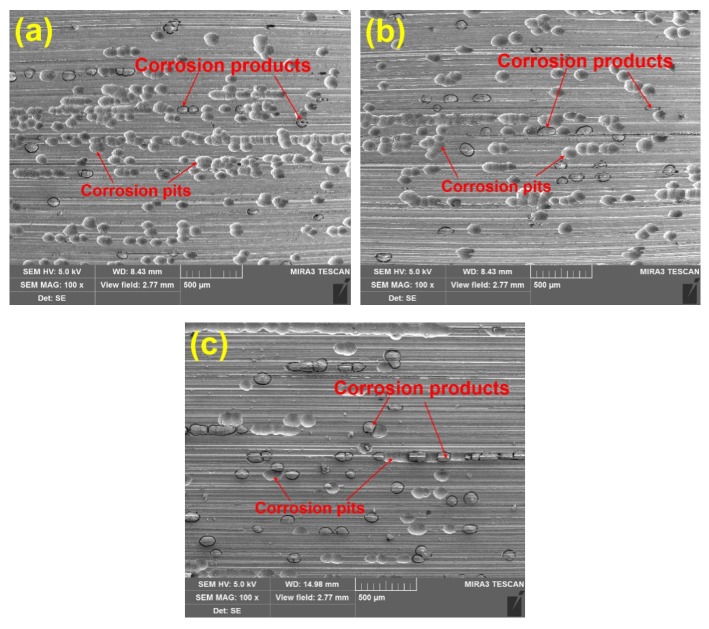
Scanning electron microscope (SEM) images of the corroded surface of samples prepared using under different particle sizes: (**a**) 36 M, (**b**) 80 M, (**c**) 120 M.

**Figure 6:**
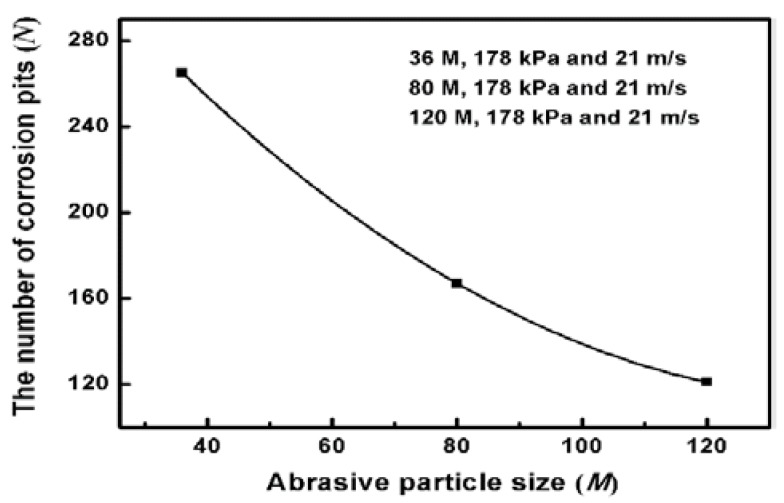
The number of corrosion pits per unit area at the corroded surface.

Ideally, electrochemical corrosion of metals should be uniform and homogeneous. Nevertheless, Inconel 718 contains many other elements to improve its mechanical properties, resulting in non-uniformity and inhomogeneity. To observe the corrosion morphology, the corroded surfaces of the ground specimens are investigated by SEM equipped with EDS to determine the change of alloying elements after the measurement of potentiodynamic polarization curves. Figure 5 shows SEM images of the corroded surface under different abrasive particle sizes. There are many corrosion pits and corrosion products with a size of about 50 μm, which indicates serious corrosion in the surface of specimens. To clearly compare the degree of corrosion behavior, the average number of corrosion pits per unit area is counted. Twenty different regions are chosen to calculate the average number of corrosion pits. The results are presented in Figure 6. It can be seen that the number of corrosion pits has an obvious decreasing trend as the abrasive particle size decreases. It can be concluded that the size reduction of abrasive particle facilitates the improvement of corrosion performance.

**Figure 7:**
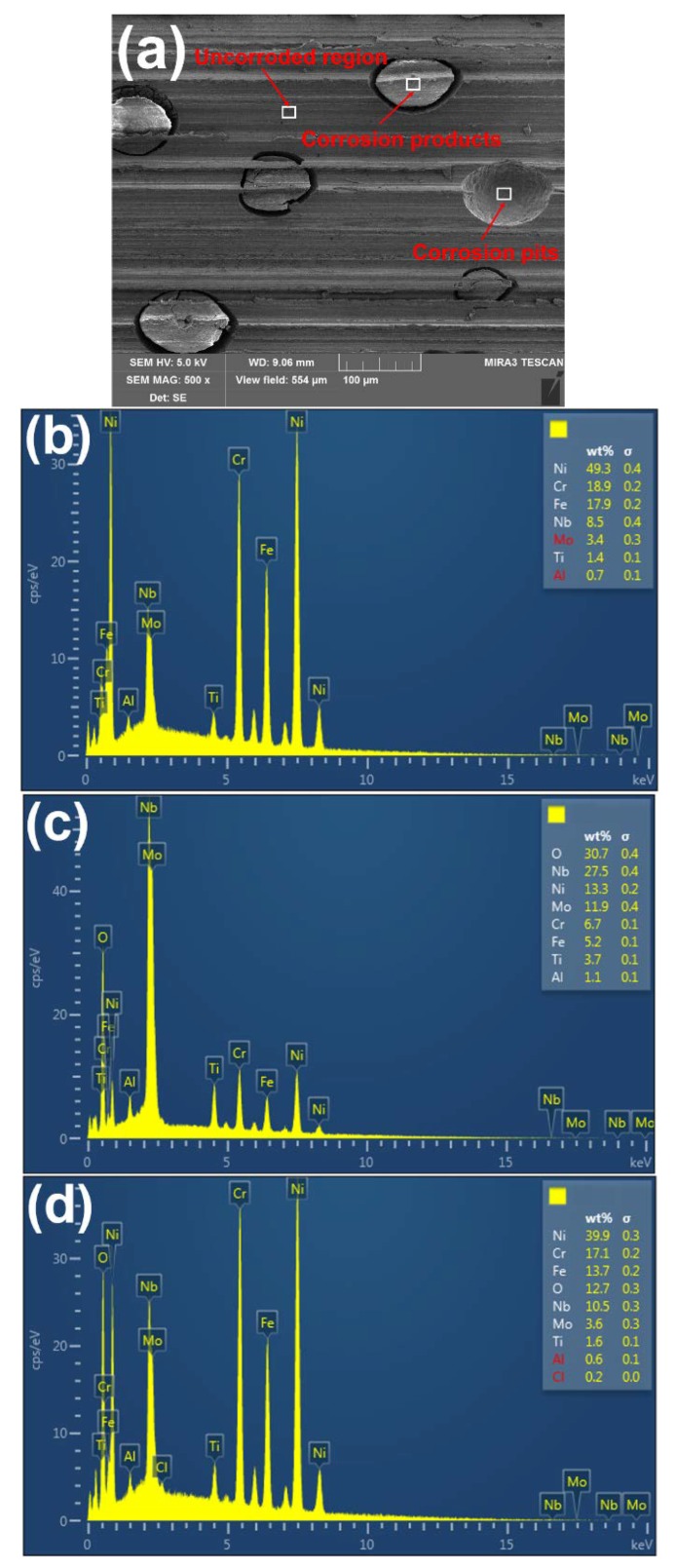
SEM and energy dispersive X-ray spectroscopy (EDS) results for the corroded specimens: (**a**) SEM image; (**b**) Uncorroded surface; (**c**) Corrosion products; (**d**) Corrosion pits.

To further investigate the corrosion behavior of the Inconel 718 under robotic belt grinding system, the change of alloying elements on the corroded surface are determined by EDS. Figure 7 shows the EDS results for different regions (uncorroded region, corrosion products, and corrosion pits) on the corroded surface. Compared with the uncorroded region and the corrosion pits, the content of Nb and O is the highest in corrosion products, which is similar to the previous results [6]. There might exist Nb-rich regions in the corrosion products. The boundary of the Nb-rich region was firstly eroded. The content of Nb and O in the corrosion pits decreases with the shedding of corrosion products.

### 3.3. Mass Loss Analysis

**Figure 8:**
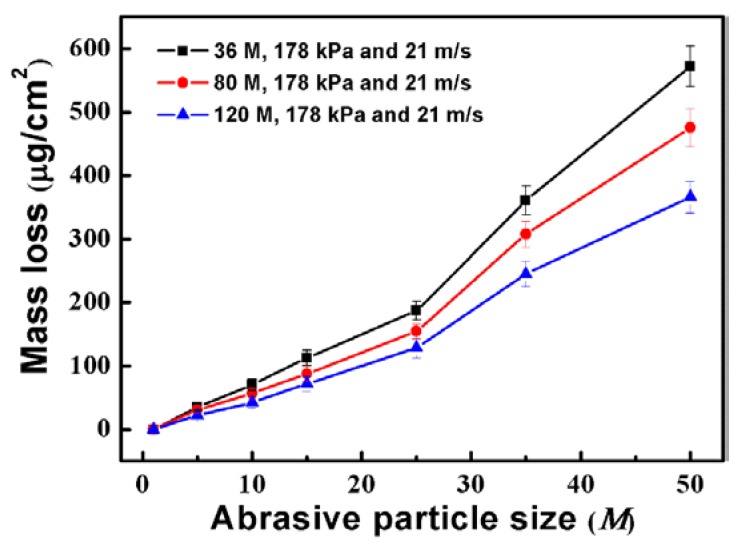
Average mass loss of the specimens after immersion for 1, 5, 10, 15, 25, 35, and 50 days.

Figure 8 shows the average mass loss of the specimens ground by different abrasive particles after different immersion times. The average mass loss of the specimens increases with the extension of immersion time. It is also found that the average mass loss decreases with the decrease of the abrasive particle size. The results indicate that the corrosion resistance of the ground surface is improved with the decrease of abrasive particle size.

### 3.4. Surface Roughness Influence

**Figure 9:**
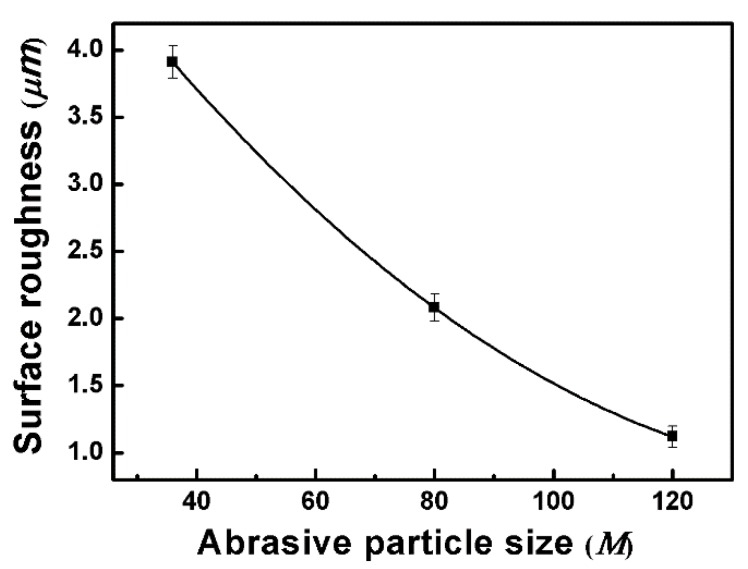
Surface roughness of treated specimens as a function of different abrasive particle sizes.

In order to further elucidate the influencing factors of corrosion resistance on nickel-based superalloys under robotic belt grinding, the surface roughness was tested. The average roughness (Ra) is measured with 2D stylus profiler over a length of 6 mm. The surface roughness of treated specimens as a function of different abrasive particle size is presented in Figure 9. There is an obviously monotonous decrease with the decrease of abrasive particle size. This is due to different surface ablation of materials during the grinding process. The larger the abrasive particle size is, the greater surface ablation and the higher surface roughness. The corrosion behavior is accelerated by the presence of large peaks and valleys which yields a larger ground surface area [34]. A larger surface area derived from higher roughness facilitates the diffusion of the electrolyte and promotes the corrosion rate [17], which is consistent with the improvement of corrosion performance as the abrasive particle size decreases. Moreover, the rough surface is harmful to the formation of the passive film [35], which is a protective film of the parts.

### 3.5. Residual Stress Analysis

**Figure 10:**
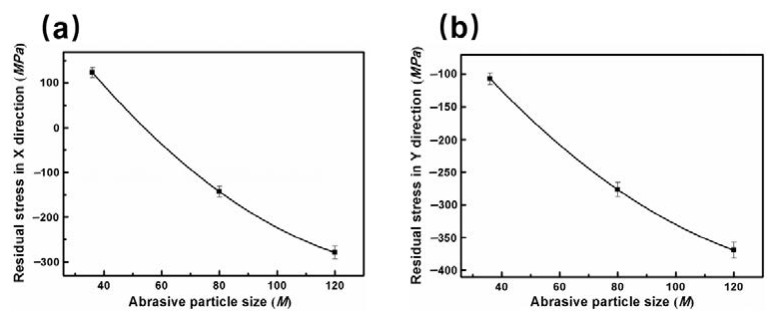
The relationship between surface residual stress and abrasive particle size: (**a**) X direction and (**b**) Y direction.

Residual stress is also a significant factor affecting the corrosion property in the grinding process [36]. During processing, metals often form part deformation and non-uniform stress. In grinding process, the residual stress forms on the specimen surface mainly due to the plastic deformation and temperature change during grinding process [37]. Normally, the corrosion occurs easily in high tensile stress and high energy state [38]. A considerable residual stress occurs in ground metallic structure [39]. Based on the importance of stress, the surface residual stress were tested in two directions (the grinding direction is defined as X and material flowing direction is defined as Y) under different abrasive particle sizes, as depicted in Figure 10. The residual stresses in both directions exhibit a similar change trend. As the abrasive particle size decreases, the residual stress in the X direction changes from the tensile stress to the compressive stress. The residual stress in the Y direction is shown as the compressive stress, and the compressive stress gradually increases with the decrease of abrasive particle size.

The effect of residual stress on corrosion performance is similar to that of stress corrosion. In stress corrosion, the metal exerts brittle cracking below yield strength in the specific corrosive medium and the tensile stress (including the applied stress and residual stress) [40]. The open circuit potential is always lower in the tensile stress concentration zone which acts as the anode of a corrosive cell. However, the compressive stress is beneficial to the formation of the passive film and enhances the corrosion resistance of the specimen surface [41,42]. The specimen surface ground by abrasive particle size of 120 M with the largest comprehensive residual stress in both directions has the best corrosion performance. This further consolidates our research result. 

### 3.6. Electrochemical Dissolution Behavior

**Figure 11:**
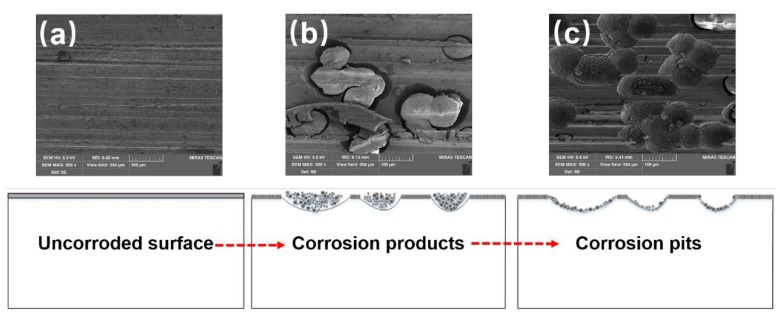
Schematic of the electrochemical dissolution behavior of Inconel 718 ground by robotic belt grinding system: (**a**) Uncorroded surface, (**b**) Corrosion products, (**b**) Corrosion pits.

Based on the results and discussion above, a plausible electrochemical dissolution behavior for Inconel 718 ground by the robotic belt grinding system can be schematically illustrated, as shown in Figure 11. The oxidation film has a porous structure which provides many natural active sites for corroding [43], and the dissolution process predominantly begins from the defective pores of the oxide film, as shown in Figure 11a. It is reasonable that the open circuit electrochemical potential of Nb is more negative, compared with other elements of Inconel 718. The Nb-rich regions occur at the defective pores of the oxide film during the corrosion process. Some corrosion products are generated in this regions. As the corrosion process continues, the dissolution occurs on the boundary between the Nb-rich region and nickel matrix according to the steep increase of current in Figure 2. The grooves are generated at the boundary which is in a high energy state and easily corroded. This is similar to the traditional pitting process [44]. With the further extension of corrosion time, the groove deepens due to the potential difference and high stress concentration, as presented in Figure 11b. With the corrosion process evolving, more products accumulate on the specimen surface and then shed into the electrolyte, leaving a large number of corrosion pits. During the shedding process, undissolved components is peeled off with the removal of the corrosion products [6]. Finally, the corrosion products shed to the electrolyte leaving the corrosion pits, as displayed in Figure 11c. 

In this experiment, the specimen surface ground by abrasive particle size of 120 M obtains the smallest roughness and the largest compressive residual stress. The smallest roughness generates a small corrosion surface area and the largest compressive residual stress protects corrosion products from shedding to electrolyte. Moreover, a small roughness can reduce the stress concentration. The combination of these factors ultimately leads to the best corrosion performance. The results further validate the electrochemical dissolution behavior of Inconel 718.

## 4. Conclusions

This paper has focused on the electrochemical corrosion behavior of Inconel 718 after robotic belt grinding. From the analysis above, the following conclusions can be made:

1. The corrosion resistance of the specimen surface improves remarkably with the decrease of surface roughness and residual stress, which result from the abrasive particle size.
2. Corrosion of Inconel 718 ground by the robotic belt grinding system proceeds from oxide film defect occurrence and Nb-rich region formation, to corrosion product generation. Then the corrosion products shed into the electrolyte due to the dissolution of the boundary, leaving a large number of corrosion pits.
3. The small roughness reduces the corrosion surface area and oxide film defect, increasing the resistance to corrosion product formation. In addition, the compressive residual stress can impede the exfoliation of corrosion products. This reasonably explains that small abrasive particle size improves the corrosion performance by producing small roughness and compressive residual stress.

This work provides significant insights in understanding of corrosion behavior of Inconel 718 treated by robotic belt system, and analyzing the influencing factors including surface roughness and residual stress. Further research in precision robotic grinding of nickel-based superalloys will revolve around modelling and optimal control of the process parameters to achieve desired properties. 

## Figures and Tables

**Table 1:** Chemical compositions of Inconel 718 (wt %).

C	Si	Mn	Al	Co	Ti	Mo	Nb	Cr	Ni	Fe
0.05	0.23	0.25	0.43	0.65	0.9	2.86	5.25	18.2	52.8	Balance

**Table 2:** Tafel polarization parameters of the ground specimens with different abrasive particle sizes.

Particle Size (M)	E_corr_ (mV vs. SCE)	I_corr_ (μA/cm^2^)
36	−800 (± 13)	35.74 (± 2.5)
80	−680 (± 9)	10.64 (± 1.2)
120	−627 (± 11)	5.28 (± 0.6)

## References

[1] González H., Pereira O., Asier Fernández-Valdivielso A., López de Lacalle L., Calleja A. (2018). Comparison of Flank Super Abrasive Machining vs. Flank Milling on Inconel^®^ 718 Surfaces. Materials.

[2] Zhao J., Liu Z., Shen Q., Wang B., Wang Q. (2018). Investigation of Cutting Temperature during Turning Inconel 718 with (Ti, Al) N PVD Coated Cemented Carbide Tools. Materials.

[3] Wang B., Liu Z., Hou X., Zhao J. (2018). Influences of Cutting Speed and Material Mechanical Properties on Chip Deformation and Fracture during High-Speed Cutting of Inconel 718. Materials.

[4] Khan M.A., Sundarrajan S., Natarajan S., Parameswaran P., Mohandas E. (2014). Oxidation and hot corrosion behavior of nickel-based superalloy for gas turbine applications. Mater. Manuf. Processes.

[5] Karthik D., Swaroop S. (2017). Laser peening without coating—An advanced surface treatment: A review. Mater. Manuf. Process..

[6] Wang D., Zhu Z., Wang N., Zhu D., Wang H. (2015). Investigation of the electrochemical dissolution behavior of Inconel 718 and 304 stainless steel at low current density in NaNO_3_ solution. Electrochim. Acta.

[7] Jebaraj J.J., Morrison D.J., Suni I.I. (2014). Hydrogen diffusion coefficients through Inconel 718 in different metallurgical conditions. Corros. Sci..

[8] Karthik D., Swaroop S. (2017). Laser shock peening enhanced corrosion properties in a nickel based Inconel 600 superalloy. J. Alloys Compd..

[9] Huang C., Wang T., Han W., Lee C. (2007). A study of the galvanic corrosion behavior of Inconel 718 after electron beam welding. Mater. Phys..

[10] Khan M.A., Prasad N.R., Krishnan S.N., Raja S.K., Jappes J.W., Duraiselvam M. (2017). Laser-treated austenitic steel and nickel alloy for human implants. Mater. Manuf. Process..

[11] Akyol A., Algul H., Uysal M., Akbulut H., Alp A. (2018). A Novel Approach for Wear and Corrosion Resistance in the Electroless Ni-P-W alloy with CNFs Co-Depositions. Appl. Surf. Sci..

[12] Narayanan B.K., Duraiselvam M., Natarajan S., Anaz Khan M. (2017). Laser material processing of nickel superalloy for improved erosion resistance. Mater. Manuf. Process..

[13] Arrizubieta J., Cortina M., Ruiz J., Lamikiz A. (2018). Combination of laser material deposition and laser surface processes for the holistic manufacture of inconel 718 components. Materials.

[14] Chen X., Devanathan R., Fong A.M. (2002). Advanced Automation Techniques in Adaptive Material Processing.

[15] Zhang X., Chen H., Xu J., Song X., Wang J., Chen X. (2018). A novel sound-based belt condition monitoring method for robotic grinding using optimally pruned extreme learning machine. J. Mater. Process. Technol..

[16] Chen J., Chen H., Xu J., Wang J., Zhang X., Chen X. (2018). Acoustic signal-based tool condition monitoring in belt grinding of nickel-based superalloys using RF classifier and MLR algorithm. Int. J. Adv. Manuf. Technol..

[17] Pradhan D., Mahobia G.S., Chattopadhyay K., Singh V. (2018). Effect of surface roughness on corrosion behavior of the superalloy IN718 in simulated marine environment. J. Alloys Compd..

[18] Wang J., Xu J., Wang X., Zhang X., Song X., Chen X. (2018). A comprehensive study on surface integrity of nickel-based superalloy Inconel 718 under robotic belt grinding. Mater. Manuf. Process..

[19] Tressia G., Penagos J., Sinatora A. (2017). Effect of abrasive particle size on slurry abrasion resistance of austenitic and martensitic steels. Wear.

[20] Turnbull A., Mingard K., Lord J., Roebuck B., Tice D., Mottershead K., Fairweather N., Bradbury A. (2011). Sensitivity of stress corrosion cracking of stainless steel to surface machining and grinding procedure. Corros. Sci..

[21] Klocke F., Soo S.L., Karpuschewski B., Webster J.A., Novovic D., Elfizy A., Axinte D.A., Tönissen S. (2015). Abrasive machining of advanced aerospace alloys and composites. CIRP Ann. Manuf. Technol..

[22] Saha P.K. (2016). Aerospace Manufacturing Process..

[23] Liu F., Wang X., Zhou B., Huang C., Lyu F. (2018). Corrosion Resistance of 2060 Aluminum–Lithium Alloy LBW Welds Filled with Al-5.6 Cu Wire. Materials.

[24] Tonpe S., Kamachi Mudali U. (2017). Effect of thermomechanical process on microstructural evolution, mechanical and corrosion properties of zircaloy-4 tubes of mock-up dissolver vessel. Mater. Manuf. Process..

[25] Liu X., Yin M., Zhang S., Wei H., Liu B., Du H., Hou L., Wei Y. (2018). Corrosion Behavior of the As-Cast and As-Solid Solution Mg-Al-Ge Alloy. Materials.

[26] De Vito E., Marcus P. (1992). XPS study of passive films formed on molybdenum-implanted austenitic stainless steels. Surf. Interface Anal..

[27] Chai Z., Jiang C., Zhu K., Zhao Y., Wang C., Cai F., Chen M., Wang L. (2016). Pretreatment Behaviors and Improved Corrosion Resistance for Cu/Co-Ni-Cu Coating Electrodeposition on Magnesium Alloy. J. Electrochem. Soc..

[28] Ma C., Han E.-H., Peng Q., Ke W. (2018). Effect of polishing process on corrosion behavior of 308L stainless steel in high temperature water. Appl. Surf. Sci..

[29] Shi Y., Pan Q., Li M., Huang X., Li B. (2014). Effect of Sc and Zr additions on corrosion behaviour of Al–Zn–Mg–Cu alloys. J. Alloys Compd..

[30] Dominguez-Crespo M.A., Torres-Huerta A.M., Rodil S., Ramírez-Meneses E., Suárez-Velázquez G., Hernández-Pérez M. (2009). Effective corrosion protection of AA6061 aluminum alloy by sputtered Al–Ce coatings. Electrochim. Acta.

[31] Jiang B., Jiang S., Ma A., Zheng Y. (2014). Effect of heat treatment on erosion-corrosion behavior of electroless Ni-P coatings in saline water. Mater. Manuf. Process..

[32] Kim Y.-S., Park J., An B.-S., Lee Y., Yang C.-W., Kim J.-G. (2018). Investigation of Zirconium Effect on the Corrosion Resistance of Aluminum Alloy Using Electrochemical Methods and Numerical Simulation in an Acidified Synthetic Sea Salt Solution. Materials.

[33] Pillis M.F., Geribola G.A., Scheidt G., de Araújo E.G., de Oliveira M.C.L., Antunes R.A. (2016). Corrosion of thin, magnetron sputtered Nb2O5 films. Corros. Sci..

[34] Sivakumar B., Pathak L.C., Singh R. (2017). Role of surface roughness on corrosion and fretting corrosion behaviour of commercially pure titanium in Ringer’s solution for bio-implant application. Appl. Surf. Sci..

[35] Balusamy T., Narayanan T.S., Ravichandran K., Park I.S., Lee M.H. (2013). Influence of surface mechanical attrition treatment (SMAT) on the corrosion behaviour of AISI 304 stainless steel. Corros. Sci..

[36] Fredj N.B., Sidhom H., Braham C. (2006). Ground surface improvement of the austenitic stainless steel AISI 304 using cryogenic cooling. Surf. Coat. Technol..

[37] Xin H., Shi Y., Ning L., Zhao T. (2016). Residual stress and affected layer in disc milling of titanium alloy. Mater. Manuf. Process..

[38] Prabhakaran S., Kulkarni A., Vasanth G., Kalainathan S., Shukla P., Vasudevan V.K. (2018). Laser shock peening without coating induced residual stress distribution, wettability characteristics and enhanced pitting corrosion resistance of austenitic stainless steel. Appl. Surf. Sci..

[39] Ding W., Zhang L., Li Z., Zhu Y., Su H., Xu J. (2017). Review on grinding-induced residual stresses in metallic materials. Int. J. Adv. Manuf. Technol..

[40] Neves F.O., Braga D.U., Silva A.S.C.D. (2015). Study of residual stresses on cold-forming metals using stress corrosion. Mater. Manuf. Process..

[41] Takakuwa O., Soyama H. (2015). Effect of residual stress on the corrosion behavior of austenitic stainless steel. Adv. Chem. Eng. Sci..

[42] Ralston K., Birbilis N. (2010). Effect of grain size on corrosion: A review. Corrosion.

[43] Ghosh S., Dey G., Dusane R., Grover A. (2006). Improved pitting corrosion behaviour of electrodeposited nanocrystalline Ni–Cu alloys in 3.0 wt. % NaCl solution. J. Alloys Compd..

[44] Du J., Ding D., Zhang W., Xu Z., Gao Y., Chen G., Chen W., You X., Chen R., Huang Y. (2017). CeLa enhanced corrosion resistance of Al-Cu-Mn-Mg-Fe alloy for lithium battery shell. Appl. Surf. Sci..

